# Autoimmune and Non-Autoimmune Comorbidities in Myasthenic Patients of East-European Descent: A Case–Control Study

**DOI:** 10.3390/jcm13082273

**Published:** 2024-04-14

**Authors:** Cristina Georgiana Croitoru, Mariana Pavel-Tanasa, Dan Iulian Cuciureanu, Diana Nicoleta Hodorog, Petru Cianga

**Affiliations:** 1 I Neurology Clinic, “Prof. Dr. Nicolae Oblu” Emergency Clinical Hospital, 700309 Iași, Romania; 2 Department of Immunology, “Grigore T. Popa” University of Medicine and Pharmacy, 700115 Iași, Romania; mariana.pavel-tanasa@umfiasi.ro; 3 Department Medical III, Discipline of Neurology, “Grigore T. Popa” University of Medicine and Pharmacy, 700115 Iași, Romania

**Keywords:** myasthenia gravis, comorbidity, chronic inflammation, granulocyte-to-lymphocyte ratio

## Abstract

**Background:** As the life expectancy of patients with myasthenia gravis (MG) is improving, so the number of comorbidities continues to rise, with a potentially significant impact on the overall morbidity and mortality. The main aim of the study was to assess comorbidities of MG in a group of patients of East-European descent. **Methods:** We retrospectively compared 185 adult myasthenic patients with 895 sex- and age-matched controls, admitted from January 2013 to December 2021. **Results:** Of these patients, 60% had late-onset MG (LOMG), with a clear predominance of women in both the LOMG and early-onset (EOMG) types; and 23.8% of the patients had a radiological description consistent with thymoma. All myasthenic patients had at least one comorbidity; 20 (10.8%) of the patients associated at least one autoimmune comorbidity. Obesity (*p* < 0.01), type 2 diabetes (*p* < 0.0001), cerebrovascular diseases (*p* < 0.0001), essential hypertension (*p* < 0.01), and cardiac arrythmias (*p* < 0.0001) were more frequent in patients than in the control group. The granulocyte-to-lymphocyte ratio was higher in the myasthenic patients compared to the controls (*p* < 0.01 for LOMG). **Discussion:** We, thus, suggest a common chronic low-grade inflammatory background as a possible connection between MG subtypes and some of these apparently unconnected comorbidities. **Conclusions:** The East-European origin of the patients offered a different social and cultural angle of a disease studied mainly on populations of West-European and Asian descent.

## 1. Introduction

Myasthenia gravis (MG) is a rare neuroimmune disorder in which fluctuating skeletal muscle fatigability is the consequence of dysfunction at the neuromuscular junction (NMJ) caused by the destruction of the complex architecture of the postsynaptic component [1]. Presented in a synthetical manner, MG is mediated by autoantibodies which are produced by B cells (B) stimulated by T cells (T), and target various postsynaptic structures such as the acetylcholine receptor (AchR), muscle-specific receptor tyrosine kinase, low-density lipoprotein related protein 4, or agrin [2,3,4]. There are various criteria used to individualize several myasthenic phenotypes which may vary in terms of therapeutical management and outcome. One of the most frequently used criteria is age at onset lower or higher than 50 years, which segregates MG into an early-onset type (EOMG) usually associated with women who have thymic hyperplasia, and a late-onset form (LOMG) which predominates in men with thymic atrophy [5]. When MG is associated with thymoma, a paraneoplastic subtype emerges, in 10–15% of cases [6].

From the novel molecular perspective, autoimmune MG is intimately connected to chronic inflammation at both of its essential immunopathogenic sites: the thymus as the origin of autoimmunity and the NMJ as the target of this autoimmunity [7]. In the past two decades, the attention of the scientific community has switched towards several easy-accessible biomarkers derived from the absolute numbers of leucocytes and platelets in the peripheral blood and their potential role in monitoring systemic inflammation in several diseases including MG [8]. The main justification of their use relies on the fact that combined parameters are much more stable than single-cell parameters [9]. From these, the blood neutrophil-to-lymphocyte ratio (NLR), systemic immune–inflammation index, and platelet-to-lymphocyte ratio were discovered at higher levels in myasthenic patients, with the latter being a possible independent predictor for respiratory failure [10]. The granulocyte-to-lymphocyte ratio (GLR) offers a potential wider picture about systemic inflammations; however, there are few studies dedicated to its prognostic role in various conditions characterized by low-grade systemic inflammation, most of them oncological [11,12].

Although the etiology and immunopathology of MG continue to be dissected throughout the scientific community, there are few data regarding the comorbidities of these autoimmune diseases and their impact towards the overall morbidity and mortality of the myasthenic patients [13,14]. As the life expectancy of myasthenic patients continues to improve due to perfecting the diagnostic techniques and treatment possibilities, it is an inevitable fact that the number of comorbidities, both autoimmune and non-autoimmune, continues to rise. Since most of the associated diseases cited, from autoimmune disorders to metabolic and cardiovascular disorders, contribute to a poorer prognosis in myasthenic patients, a vast knowledge of possible comorbidities and their management is imperious in individuals diagnosed with MG [15].

The main aim of the current study was to assess the comorbidities of MG in a relatively large study group of myasthenic cases from a neurology clinic representative for the north-eastern region of Romania. Moreover, we propose a common chronic low-grade inflammatory background as a possible connection between MG subtypes and apparently unconnected comorbidities such as endocrinological, cardiovascular, and metabolic diseases.

## 2. Materials and Methods

### 2.1. Study Population

The current study was of an analytic retrospective design as we compared a group of 185 adult myasthenic patients with a control group of 895 sex- and age-matched individuals, admitted in the First Neurology Clinic of the “Prof. Dr. N. Oblu” Emergency Clinical Hospital, Iași, Romania, in a nine-year period, between 1 January 2013–31 December 2021. 

After excluding patients with other myasthenic syndromes (18) or unconfirmed diagnosis (6), 191 patients checked all positive diagnostic criteria for MG. Six patients with incomplete classical laboratory biomarkers were excluded. The remaining 185 patients were included in the study group regardless of comorbidities or classes of anti-myasthenic treatment undertaken. All patients included underwent chest computer tomography (CT) in order to assess thymus morphology. The positive diagnostic criteria for MG considered overlapped the ones stipulated in international guidelines: clinical manifestations highly suggestive for MG and minimum one paraclinical criteria from immunologic testing (elevated titres of acetylcholine receptor antibodies and/or muscle specific tyrosine kinase receptor antibodies) and/or electrophysiologic testing (characteristic decrement over 10% of compound muscle action potential between the first and the fifth stimulus at slow rate electrical stimulation of the nerve and/or prolonged jitter at single-fibre electromyography) [6,16]. We included both new cases, diagnosed per primam during the nine-year time frame and also chronic cases defined as patients diagnosed before the beginning of the study.

The control group comprised 895 patients admitted in the same time interval in the neurological and neurosurgical clinics of the same hospital for unrelated diseases of the central and peripheral nervous system. For matching the mean age of the EOMG and LOMG groups, the control group was further divided in two main subgroups: one group younger than 50 years (300 females, 177 males)—average age of 39, and another group older than 50 years (296 females, 122 males)—average age of 62 (Figure S1).

### 2.2. Patient Clinical and Paraclinical Data

All clinical, laboratory, and radiological data were collected from the electronic records and patients’ medical files. Numerous variables were included: sex, age at admission, age at onset of MG, disease duration, Myasthenia Gravis Foundation of America (MGFA) classification at discharge, autoimmune and non-autoimmune comorbidities, classical blood laboratory biomarkers (complete blood count, glucose, total cholesterol and fractions, and triglycerides—TG, urea, creatinine, and liver transaminases), and morphology of the thymus [17]. Data regarding comorbidities were collected using the International Statistical Classification of Diseases and Related Health Problems—10 (ICD-10) codification.

The clinical MG subtypes considered were LOMG/EOMG and ocular (OMG) versus generalized (GMG) MG defined as Class I, respectively, as classes II–V MGFA [17].

Blood samples were obtained by venipuncture à jeun at admission and all routine laboratory biomarkers mentioned above were measured by standard methods in the hospital’s laboratory: flow cytometry for complete blood count and spectrophotometry for total cholesterol and cholesterol fractions, TG, glucose, urea, creatinine, and liver transaminases. GLR was calculated by dividing the absolute number of granulocytes by the absolute number of lymphocytes [11].

Thymus morphology was assessed by chest computed tomography (CT) performed during respiratory pause after deep inspiration. Intravenous radiological contrast was administered in most of the cases, especially in those with a high suspicion of thymoma. In accordance with consecrated radiological terms, we systematized all 185 radiological descriptions into three entities: thymic focal mass (TFM) which included aspects highly suggestive for thymoma, diffuse enlargement of thymus (DET) which included aspects highly suggestive for thymic follicular hyperplasia, and an aspect resembling that of the patient’s age (RPA) which included aspects highly suggestive for thymic atrophy or remaining thymic tissue in patients older, respectively, and younger than forty years [18].

### 2.3. Statistical Analysis

Statistical analysis was performed using Graph Pad Prism, v5 (Graph Pad Software, San Diego, CA, USA). Data are generally presented as bars with information about the mean and standard error of mean (SEM) when required. Each figure legend contains information about the significance *p* value and the corresponding statistical tests performed. The Shapiro–Wilk test was used to check for normality distribution. The parametric data were analyzed using the unpaired *t*-test and one-way ANOVA with post hoc Tukey’s multiple comparison test. For non-parametric data, the following statistical tests were applied: Mann–Whitney test (the non-parametric counterpart to unpaired *t*-test), and Kruskal–Wallis with Dunn’s multiple comparison test (the non-parametric counterpart to one-way ANOVA). Chi-squared test was used for comparing categorical variables. The associations between measured variables were performed using the Spearman’s correlation coefficients (R). Absolute R values between 0.2–0.39 were treated as weak, between 0.4–0.59 as moderate, between 0.6–0.79 as strong, and between 0.8–1.00 as very strong correlation factors. The linear regression graphs show the best-fit line with the 95% confidence band. The F-test was used to determine the level of significance, while the coefficient of determination R-squared (R2) was used as a goodness-of-fit measure for each linear regression. The *p* values less than 0.05 were considered statistically significant.

## 3. Results

### 3.1. General Characteristics of MG Subjects

**Figure 1:**
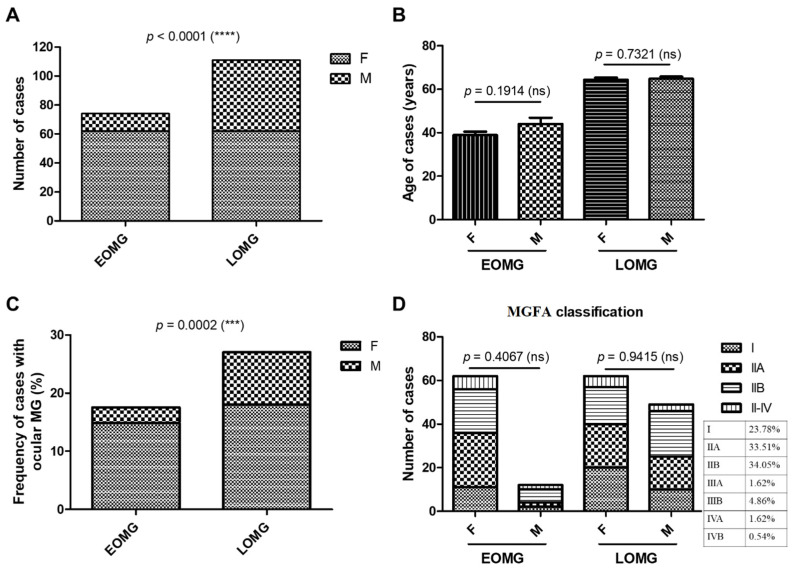
General characteristics of subjects with EOMG vs. LOMG. (**A**) Number of women and men in the two subject groups: EOMG and LOMG (**** *p* < 0.0001; chi-squared test). (**B**) Age of female and male cases in the EOMG and LOMG groups. Bars represent the mean ± SEM (ns = not significant; two-tailed unpaired *t*-test). (**C**) Prevalence of ocular MG in EOMG and LOMG cases (*** *p* < 0.001; chi-squared test). (**D**) MGFA clinical classification of EOMG and LOMG cases (ns = not significant; chi-squared-test).

Among the 185 MG cases (78 new + 107 chronic), 124 were women and 61 were men. The EOMG group comprised 40% of cases (62 women and 12 men), while the LOMG represented the rest of 60% (62 women and 49 men), with significant predominance of women in both MG groups (*p* < 0.0001, chi-squared test) (Figure 1A). In the EOMG group, the average age was 39 years for women and 44 years for men (with no significant differences), while, in the LOMG group, the mean age for both women and men was 64 (Figure 1B). The disease duration of MG ranged from 0 to 29 years (average of 5.43 years) in the EOMG group, and from 0 to 12 years (average 1.36 years) in the LOMG group. In the entire studied group, patients with generalized MG were the majority. The prevalence of ocular MG was 17.56% among early-onset cases and 27.03% among late-onset cases. The female-to-male ratio in the ocular EOMG and LOMG groups mirrored the general sex distribution (Figure 1C). Based on MGFA classification, no significant differences in disease severity were observed when comparing males and females, in both the EOMG and LOMG groups. The majority of the cases were classified as MGFA I, IIA, and IIB, the last two each representing approximately one-third of the lot (Figure 1D).

### 3.2. Associated Diseases in Myasthenic Patients

**Figure 2:**
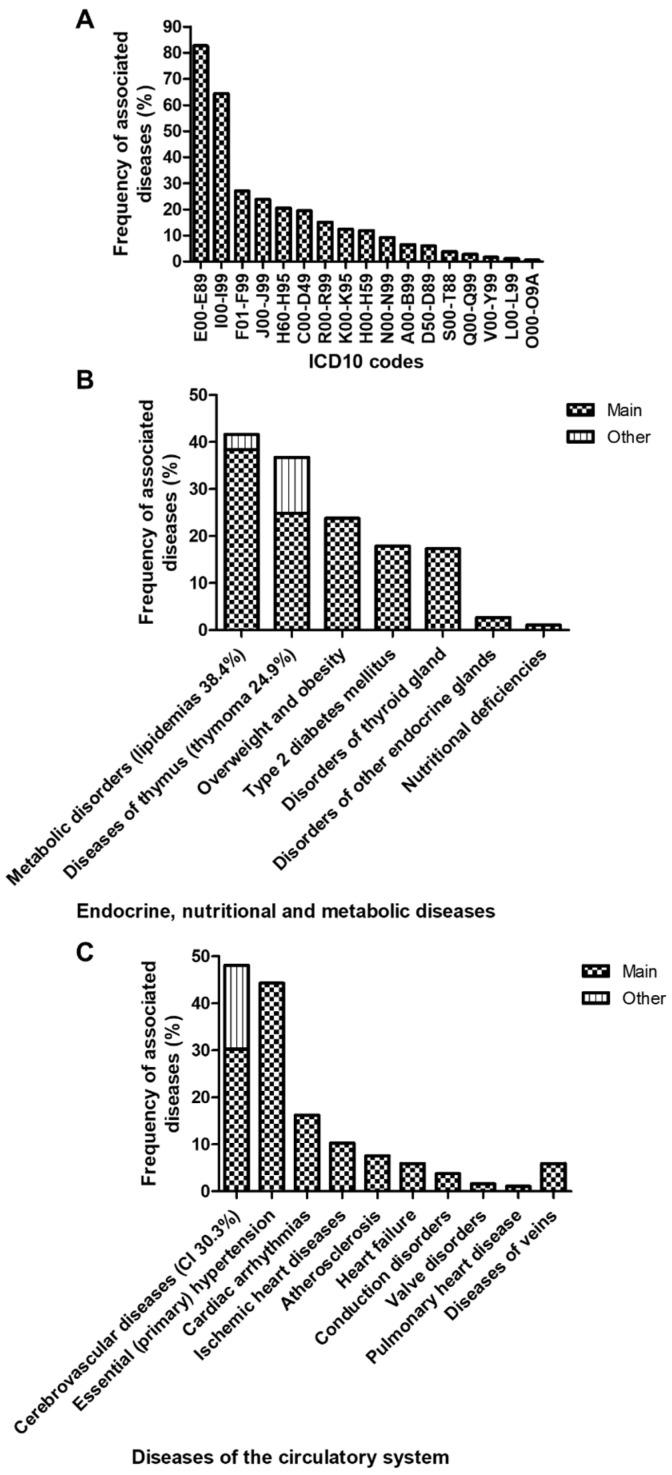
Proportion of MG cases associated with the indicated ICD-10 codes. (**A**) Proportion of MG cases associated with the indicated ICD-10 chapters. (**B**) Proportion of MG cases associated with the indicated endocrine, nutritional, and metabolic diseases. (**C**) Proportion of MG cases associated with the indicated diseases of the circulatory system. CI = cerebral infarction.

The MG cases were further investigated for comorbidities based on the ICD-10 codes stipulated in the medical records during the hospitalization period. All 185 patients within our study were associated with at least one comorbidity, thus checking seventeen out of the twenty-two internationally recognized ICD chapters. Our myasthenic patients were associated most frequently with endocrine, nutritional, and metabolic disorders (Chapter IV, codes E00-E89), and diseases of the circulatory system (Chapter IX, codes I00-I99) (Figure 2A). On the other hand, mental and behavioral disorders (codes F01-F99), diseases of the respiratory system (codes J01-J99), diseases of the ear and mastoid process (codes H60-H95), neoplasms (codes C00-D49), diseases of the digestive system (codes K00-K95), diseases of the eye and adnexa (codes H00-H59), diseases of the genitourinary system (codes N00-N99), infectious and parasitic diseases (codes A00-B99), diseases involving the immune mechanism (codes D50-D89), injury and certain other consequences of external causes (codes S00-T88 and V00-Y99), congenital malformations, deformations and chromosomal abnormalities (codes Q00-Q99), diseases of the skin and subcutaneous system (codes L00-L99) and diseases related to or aggravated by pregnancy, childbirth and puerperium (codes O00-O09A) were present, to a lesser extent (Figure 2A).

Then, 82.70% of the MG subjects were associated with endocrine, nutritional, and metabolic disorders (38.4% dyslipidemias, 24.9% thymoma, 23.8% overweight and obesity, 17.8% type 2 diabetes mellitus, and 17.3% disorders of thyroid gland; Figure 2A,B), while 64.32% of the investigated subjects presented various cardiovascular diseases (50.1% cerebrovascular diseases, 44.3% essential hypertension, 16.2% cardiac arrhythmias, 10.3% ischemic heart diseases, and 7.6% atherosclerosis; Figure 2A,C). The rest of the ICD-10 codes were identified, to a lesser extent, in less than 30% of subjects, including anxiety in 20% of myasthenic patients and depression in less than 5% (detailed in Figure S2).

**Figure 3:**
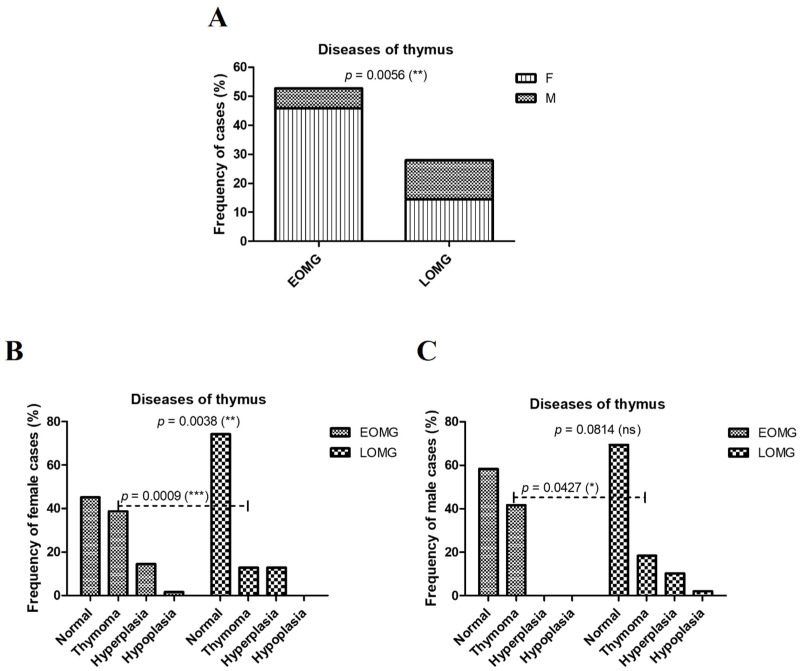
Diseases of the thymus in subjects with EOMG vs. LOMG. (**A**) Proportion of female and male MG cases with thymus disease (** *p* < 0.01; chi-squared test). (**B**) Prevalence of thymus disease (thymoma, hyperplasia, and hypoplasia) in women with EOMG and LOMG (*** *p* < 0.001, ** *p* < 0.01; chi-squared test). (**C**) Prevalence of thymus disease (thymoma, hyperplasia, and hypoplasia) in men with EOMG and LOMG (* *p* < 0.05, ns = not significant; chi-squared test).

According to ICD codification, regarding the thymus diseases (thymoma, hyperplasia, and hypoplasia) in MG cases, the prevalence was significantly higher among EOMG subjects (52.71%) compared to LOMG (27.92%, *p* = 0.0056) (Figure 3A). This difference was due to a significant two- to three-fold increase in thymoma rates among both females and males with EOMG (females: 38.7% EOMG vs. 12.9% LOMG, *p* = 0.0009; males: 41.7% EOMG vs. 18.4% LOMG, *p* = 0.0427) (Figure 3B,C).

**Figure 4:**
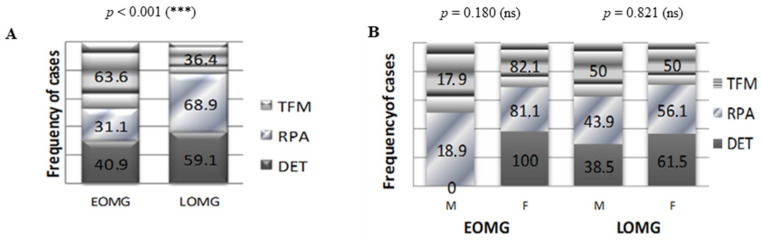
Radiological aspect of the thymus in patients with EOMG vs. LOMG. (**A**) Proportion of TFM, DET, and RPA in patients with EOMG and LOMG (*** *p* < 0.001, chi-squared test). (**B**) Proportion of radiological aspects of the thymus in women and men with EOMG (*p* = 0.180, ns = not significant; chi-squared test) and LOMG (*p* = 0.821, ns = not significant; chi-squared test).

When addressing the thymic morphology resulting from the thoracic CT description, 23.8% of the myasthenic patients studied had thymoma (TFM), 11.9% had thymic follicular hyperplasia (DET), and the rest showed a chest CT aspect consistent with their age (RPA). While thymic follicular hyperplasia was encountered with a similar frequency in both EOMG (40.9%) and LOMG (59.1%) patients, thymoma was predominant in EOMG patients (63.6%) compared to LOMG (36.4%, *p* < 0.001), and a thymic aspect in concordance with the patient’s age predominated in LOMG patients (68.9%) compared to EOMG (31.1%, *p* < 0.001, Figure 4A). No significant correlations were established between the gender of the myasthenic patients and the thymic morphology (*p* = 0.390, Table 1). Despite the fact that no statically significant correlations were encountered between thymus morphology and sex in neither of the EOMG or LOMG subgroups, in the EOMG subgroup, thymoma was more frequently encountered in women, while thymic follicular hyperplasia was encountered exclusively in women. In the LOMG subgroup, the frequency of the three thymic CT aspects was similar among women and men (Figure 4B).

### 3.3. Endocrine Comorbidities in MG Patients Versus Controls

**Figure 5:**
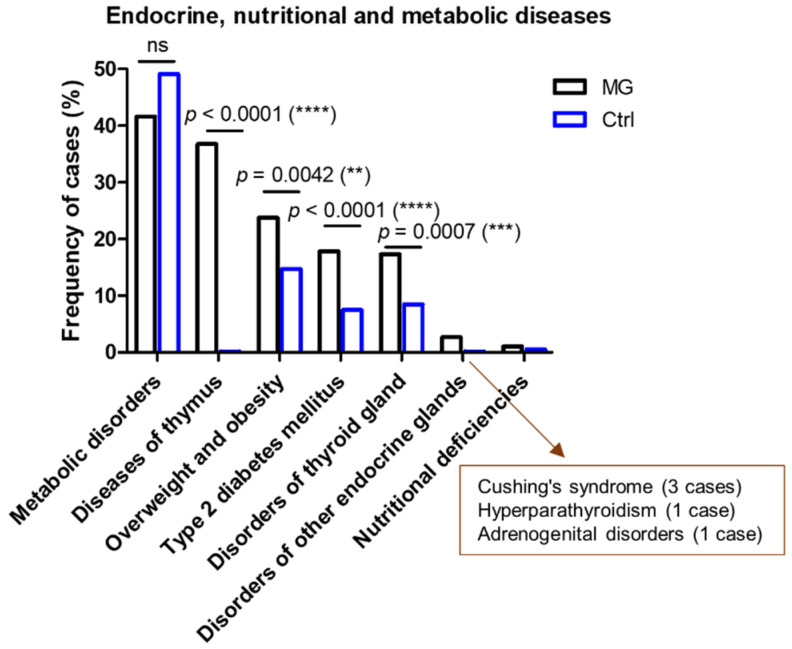
Proportion of MG and control subjects associated with the indicated endocrine, nutritional, and metabolic diseases. (**** *p* < 0.0001, *** *p* < 0.001, ** *p* < 0.01; two-sided Fisher’s exact test).

The MG cases showed a higher frequency of thyroid gland disorders (17.3% vs. 8.5%, *p* = 0.0007, two-sided Fisher’s exact test) compared to the controls. We were also able to identify, among the MG group, pathologies of other endocrine glands: three cases associated with Cushing’s syndrome, one case with hyperparathyroidism, and another one with adrenogenital disorders (Figure 5).

**Figure 6:**
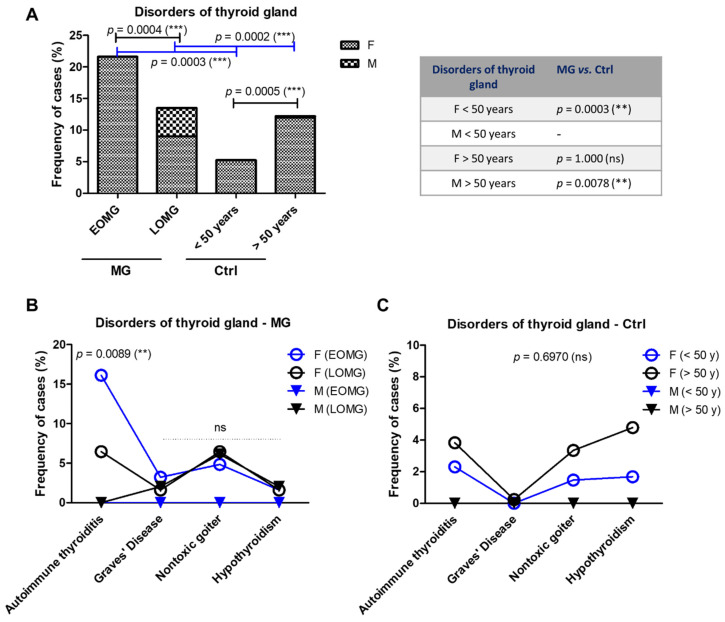
Diseases of the thyroid gland in subjects with MG vs. control group. (**A**) Proportion of MG and control subjects associated with disorders of the thyroid gland (*** *p* < 0.001, ** *p* < 0.01, ns = not significant; chi-squared test for the graphic on the left, two-sided Fisher’s exact test for the table on the right). (**B**) Prevalence of thyroid disorders (autoimmune thyroiditis, Graves’ disease, nontoxic goiter, and hypothyroidism) in women and men with EOMG and LOMG (** *p* < 0.01, ns = not significant; chi-squared test). (**C**) Prevalence of thyroid disorders (autoimmune thyroiditis, Graves’ disease, nontoxic goiter, and hypothyroidism) in the control group (ns = not significant; chi-squared test).

The prevalence of thyroid gland disorders was increased in the EOMG (21.62%) compared to the LOMG group (13.51%, *p* = 0.0004). In the control group, the trend was opposite: the younger group (<50 years) showed a lower frequency compared to the older group (>50 years): 5.24% vs. 12.20% (*p* = 0.0005) (Figure 6A). Male cases with thyroid disorders were seen only in the groups over 50 years old (both MG and controls), however, with a higher frequency among MG cases: 4.5% in LOMG compared to an extremely low value of 0.24% in the control group (*p* = 0.0078) (Figure 6A). The increased percentage of cases with thyroid disorders seen in EOMG when compared to LOMG was due to the high prevalence of chronic autoimmune thyroiditis at 16.13%, while the other thyroid pathologies showed lower frequencies: Grave’s disease, nontoxic goiter, or non-autoimmune hypothyroidism (Figure 6B). In addition, in the EOMG subgroup, chronic autoimmune thyroiditis was exclusively encountered in women (*p* = 0.0089) (Figure 6B). There were no significant differences among the percentages of females with autoimmune thyroiditis between the LOMG and control (>50 years) groups (Figure 6C).

**Figure 7:**
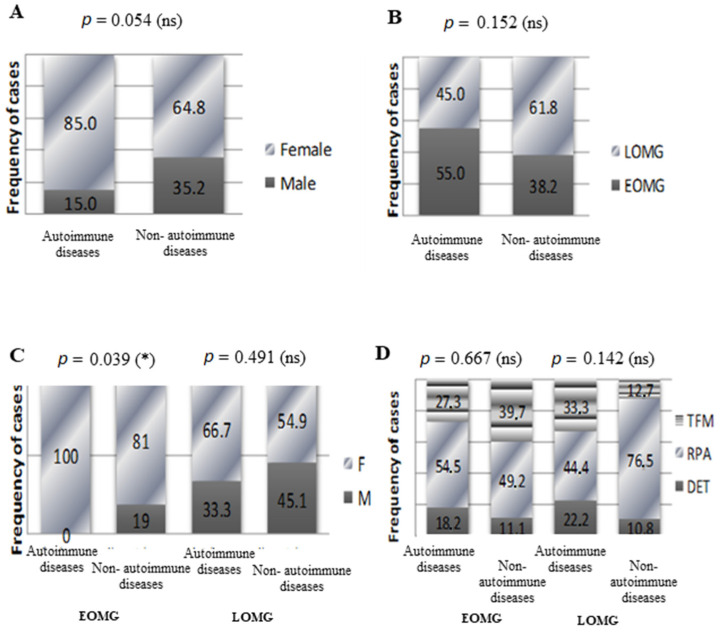
Clinical and demographical characteristics of MG patients with and without autoimmune comorbidities. (**A**) Proportion of women and men with and without autoimmune comorbidities (*p* = 0.054, ns = not significant; chi-squared test). (**B**) Proportion of patients with EOMG and LOMG with and without autoimmune comorbidities (*p* = 0.152, ns = not significant; chi-squared test). (**C**) Proportion of women and men with and without autoimmune comorbidities in the EOMG (* *p* < 0.05; chi-squared test) and the LOMG (*p* = 0.491; ns = not significant; chi-squared test) groups. (**D**) Proportion of radiological aspects of the thymus in patients with and without autoimmune comorbidities with EOMG (*p* = 0.667, ns = not significant; chi-squared test) and LOMG (*p* = 0.142, ns = not significant; chi-squared test).

As autoimmunities often tend to be associated, we were particularly interested in these comorbidities. Therefore, we divided our myasthenic lot into two subgroups according to the presence or absence of autoimmune comorbidities and, next, compared the basic clinical and demographical characteristics between them. Twenty of our patients (10.8%) presented at least one autoimmune comorbidity: chronic autoimmune thyroiditis (11 cases), Graves’ disease (3 cases), rheumatoid arthritis (3 cases), vitiligo (1 case), megaloblastic anemia (1 case), and an association of chronic autoimmune thyroiditis and rheumatoid arthritis (1 case). No statistically significant associations between overall autoimmune comorbidities and MG subgroups (LOMG vs. EOMG, and thymomatous MG vs. non-thymomatous MG) resulted for our group of patients. Irrespective of the presence or absence of autoimmune comorbidities, myasthenic women represented the majority over men (*p* = 0.054) (Figure 7A). Autoimmune comorbidities were slightly more frequent in EOMG patients (55%) when compared to LOMG (45%, *p* = 0.152) (Figure 7B). Interestingly, all autoimmune comorbidities within the EOMG subgroup were encountered exclusively in women (*p* = 0.039); hence, this situation generated the strongest statistically correlation (Figure 7C). Thymomas were slightly more frequently encountered in LOMG patients with autoimmune comorbidities (LOMG: with autoimmune comorbidities 33.3% vs. without autoimmune comorbidities 12.7%, *p* = 0.142) and in EOMG patients without autoimmune comorbidities (EOMG: with autoimmune comorbidities 27.3% vs. without autoimmune comorbidities 39.7%, *p* = 0.667) (Figure 7D). No significant associations could be established between autoimmune comorbidities and disease severity, as MGFA classes I, IIA, and IIB were the majority in both subgroups of myasthenic patients (Table 2). However, the overall tendency in terms of disease severity in myasthenic patients with associated autoimmune comorbidities when compared to myasthenic patients without other autoimmune diseases was that of a higher proportion of class IIB, followed by class I (Table 2).

### 3.4. Metabolic Comorbidities in MG Patients Versus Controls

**Figure 8:**
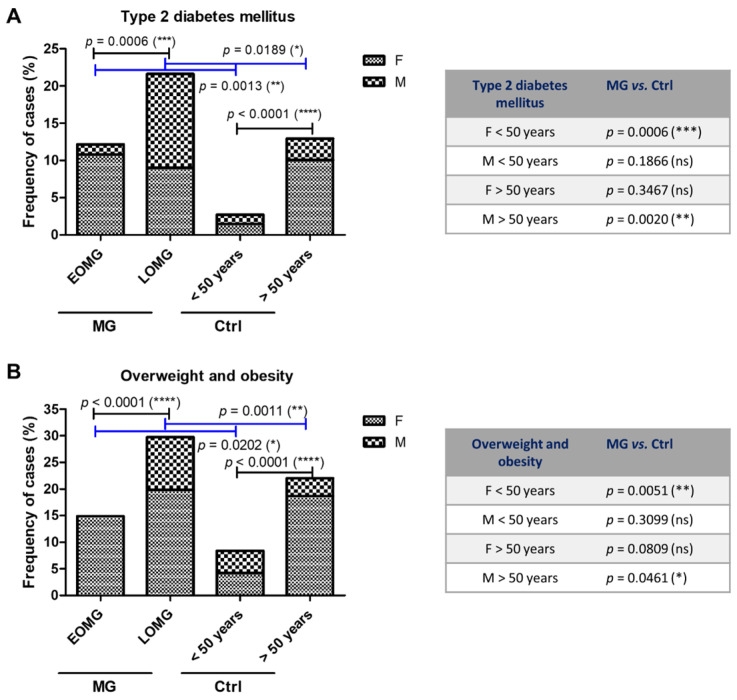
Other endocrine and metabolic disorders in subjects with MG vs. control group. (**A**) Proportion of MG and control subjects associated with type 2 diabetes mellitus (**** *p* < 0.0001, *** *p* < 0.001, ** *p* < 0.01, * *p* < 0.05, ns = not significant; chi-squared test for the graphic on the left, one-sided Fisher’s exact test for the table on the right). (**B**) Proportion of MG and control subjects associated with overweight and obesity (**** *p* < 0.0001, ** *p* < 0.01, * *p* < 0.05, ns = not significant; chi-squared test for the graphic on the left, one-sided Fisher’s exact test for the table on the right).

The proportion of cases with obesity and type 2 diabetes mellitus was double in the MG group compared to the control subjects (23.8% and 17.8% in MG vs. 14.7% and 7.5% in controls)—(Figure 5). As expected, we noticed a raised frequency of these two metabolic diseases in the LOMG group when compared to EOMG, as well as when comparing the >50 years and the <50 years control groups. However, the general prevalence of these metabolic disorders was higher in the MG groups compared to the control groups, and those differences were rather due to a rise in the frequency of female EOMG patients (*p* = 0.0006 for type 2 diabetes; *p* = 0.0051 for obesity) and the frequency of male LOMG patients (*p* = 0.0020 for type 2 diabetes, *p* = 0.0461 for obesity) (Figure 8).

### 3.5. Cardiovascular Comorbidities in MG Patients Versus Controls

**Figure 9:**
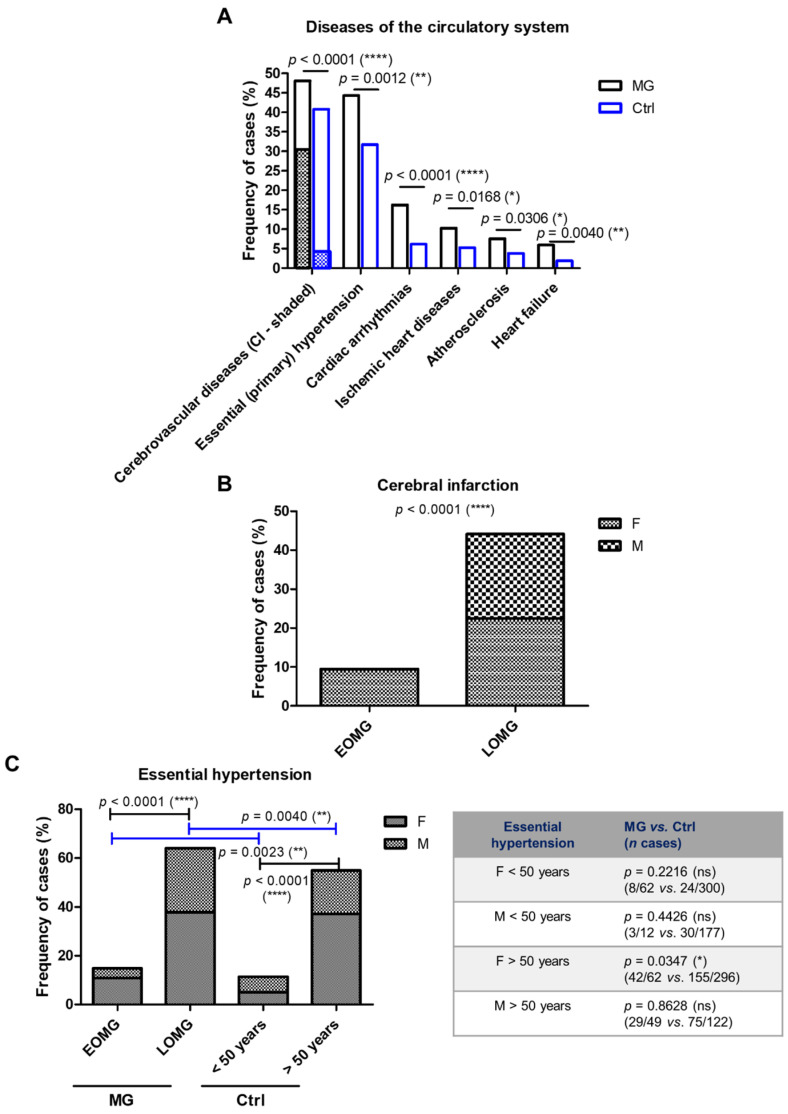
Differences in the frequencies of cerebral and cardiovascular diseases between MG and control subjects. (**A**) Proportion of MG cases and control subjects associated with various diseases of the circulatory system (**** *p* < 0.0001, ** *p* < 0.01, * *p* < 0.05; chi-squared test). (**B**) Prevalence of cerebral infarction in women and men with EOMG and LOMG (**** *p* < 0.0001; chi-squared test). (**C**) Proportion of MG and control subjects associated with essential hypertension (**** *p* < 0.0001, ** *p* < 0.01, * *p* < 0.05, ns = not significant; chi-squared test for the graphic on the left, one-sided Fisher’s exact test for the table on the right). Ci = cerebral infarction.

As the frequency of cerebral and cardiovascular diseases was over 60% among the MG cases, we, next, tested whether this high prevalence characterizes the control group. The frequencies of various cardiovascular pathologies, including the cerebral infarction (30% in the MG group vs. 5% in the control group, *p* < 0.0001), essential hypertension (44.3% MG vs. 31.7% Ctrl, *p* = 0.0012), or cardiac arrhythmias (16.2% MG vs. 6.1% Ctrl, *p* < 0.0001) were significantly higher among MG subjects when compared to the control group (Figure 9A). Furthermore, the frequency of cerebrovascular diseases from which cerebral infarction was dominant was clearly higher among the LOMG group compared to EOMG (44.1% vs. 9.5%) and evenly distributed among females and males only in the LOMG group, while, in the EOMG subgroup, this was encountered only in women (Figure 9B). Similarly, the frequency of primary hypertension was also higher among the LOMG cases, but significantly higher for both MG groups (EMOG and LOMG) when compared to the control groups (<50 years and >50 years, respectively). The differences seen between the MG patients and control subjects were, thus, actually determined by the female subgroup (Figure 9C).

### 3.6. GLR, Blood Glucose, Lipidogram, and Liver and Hepatic Function in MG Patients Versus Controls

**Figure 10:**
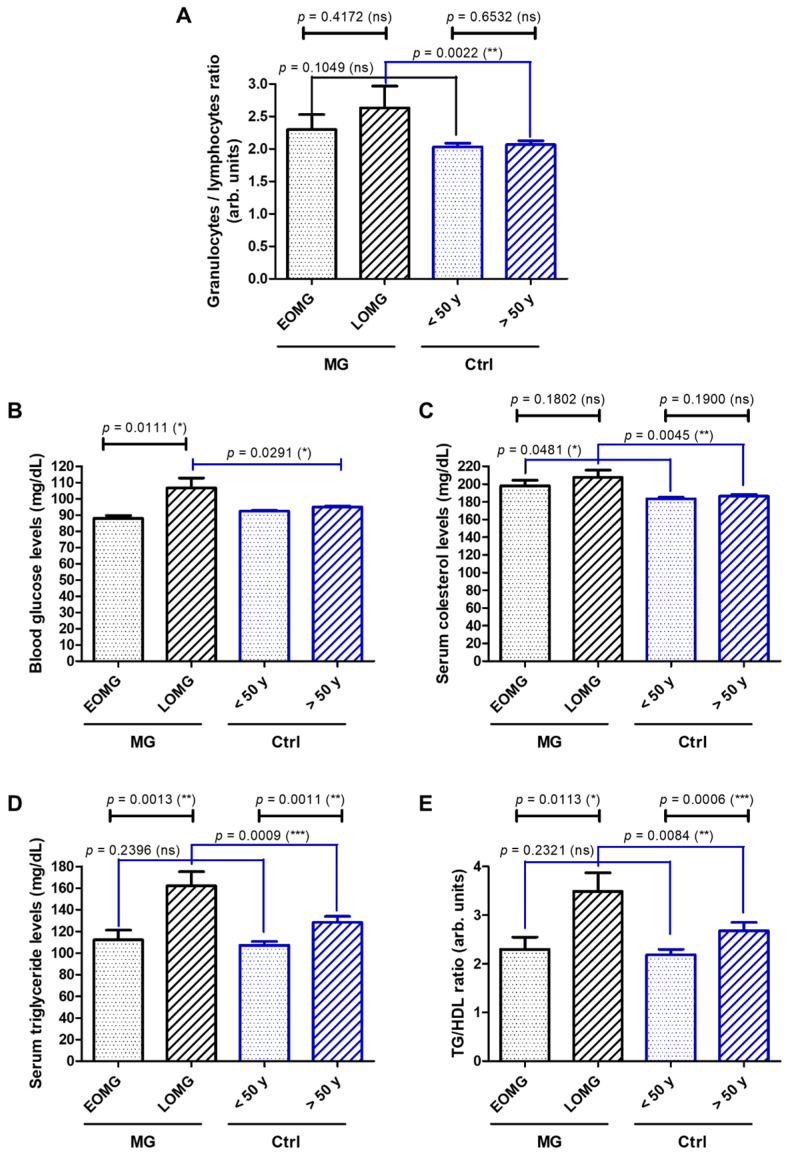
Laboratory biomarkers in MG vs. controls: (**A**) granulocyte-to-lymphocyte ratio, (**B**) blood glucose, (**C**) total cholesterol level, (**D**) triglyceride (TG) levels, (**E**) TG-to-high-density-lipoproteins (HDL) ratio (*** *p* < 0.001, ** *p* < 0.01, * *p* < 0.05, ns = not significant; one-way ANOVA for normally distributed data and Mann–Whitney test otherwise).

In order to further evaluate the aforementioned comorbidities of the myasthenic patients included in our study, we, next, investigated the potential differences between various subgroups of MG and control subjects of some laboratory biomarkers’ values that are routinely tested in our hospital. Firstly, the blood count data revealed an increased GLR in the MG subjects above 2.0 (2.3 for EOMG and 2.6 for LOMG), which was the average value observed for the control cases. The observed difference was significant only for the category of older subjects (LOMG vs. >50 years controls, *p* = 0.0022 (Figure 10A). The blood glucose was slightly higher (above 100 mg/dL) in the LOMG group compared to the counterpart controls (*p* = 0.0291) (Figure 10B). When investigating the lipid metabolism, we observed borderline high cholesterol levels (>200 mg/dL) for MG cases (both EOMG and LOMG (Figure 10C), but significantly higher TG levels and TG-to-high-density-lipoprotein-cholesterol (TG/HDL) ratios only in the LOMG group (Figure 10D,E).

**Figure 11:**
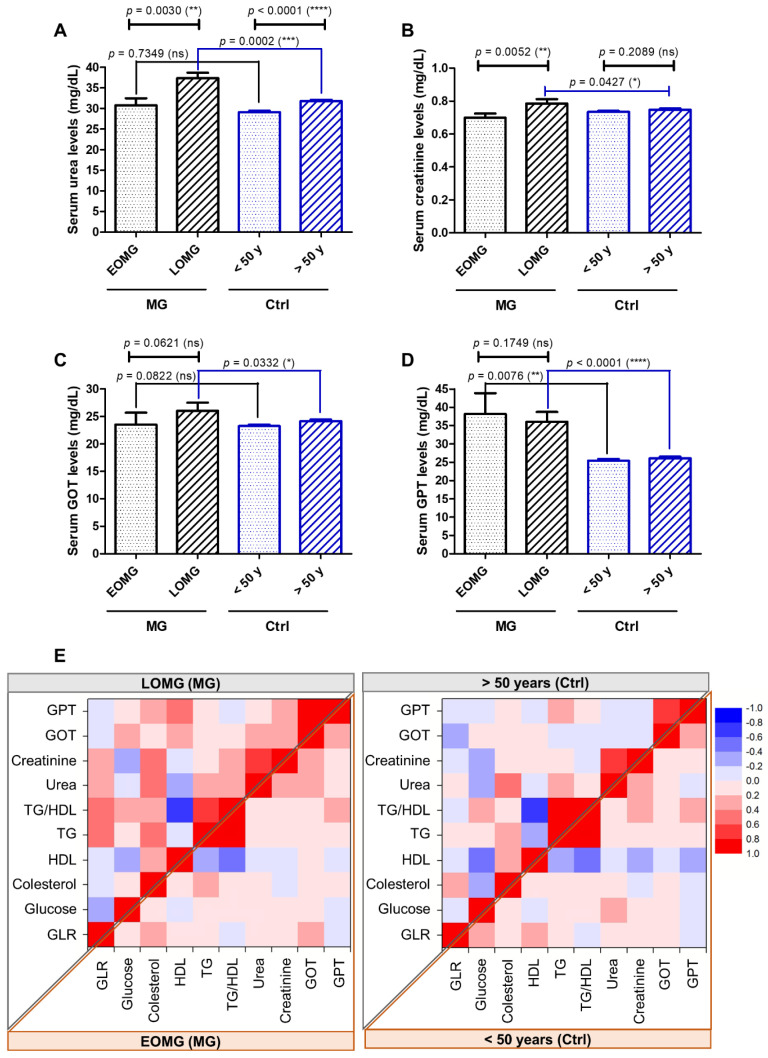
Classic diagnostic biomarkers of kidney and liver injury in MG vs. control subjects: (**A**) serum urea, (**B**) serum creatinine, (**C**) serum glutamic oxaloacetic transaminase (GOT), and (**D**) serum glutamic pyruvic transaminase (GPT) levels (**** *p* < 0.0001, *** *p* < 0.001, ** *p* < 0.01, * *p* < 0.05, ns = not significant; one-way ANOVA for normally distributed data and Mann–Whitney test otherwise). (**E**) Heat map of correlation coefficients (R) between various laboratory biomarkers among MG (**left**; LOMG—upper and EOMG—bottom panel) and control (**right**; ≥50 years—upper and <50—bottom panel) subjects (0.2–0.39: weak; 0.4–0.59: moderate; 0.6–0.79: strong; 0.8–1.0: very strong correlations).

Despite being in the normal range, the LOMG cases, compared to their counterpart controls, presented significantly higher serum levels of urea (*p* = 0.0002) (Figure 11A), creatinine (*p* = 0.0427, Figure 11B), and liver transaminases (GOT: *p* = 0.0332; GPT: *p* < 0.0001 (Figure 11C,D). When examining the potential correlations between these aforementioned laboratory biomarkers, we noticed only few and weak correlations for the control groups and the EOMG group. However, for the LOMG group, we distinguished multiple moderate and strong positive correlations between GLR and TG (or TG/HDL), between total cholesterol and urea or creatinine levels, and between glucose and TG or transaminase levels (Figure 11E).

Since, for all classes of anti-myasthenics, oral corticotherapy administered for a prolonged period especially could have been involved, at least to some extent, in the increased occurrence of obesity, diabetes, mood disorders, cardiovascular diseases, and some of the elevated hematological biomarkers, we, next, assessed the specific chronic treatments undergone by the chronic patients. Our study group consisted of 185 myasthenic patients, out of which 107 were already treated with chronic oral anti-myasthenic drugs. Further, out of these 107 patients, while only 4.67% received azathioprine, 48 received chronic oral prednisone or equivalent doses of prednisone, at an average daily dose of 24.01 mg ± 15.63 (median = 20 mg), varying from a minimum of 2.5 mg to a maximum of 60 mg per day. In order to investigate the possible impact of these medications upon various comorbidities, we compared the frequency of their occurrence between the 48 patients group and the rest of the 59 chronic patients, on one side, as well as with the 137 patients (new and chronic) that did not receive corticotherapy, on the other side. Among the 48 patients who received chronic oral corticotherapy, over 40% were associated with dyslipidemias and hypertension, while less than a quarter presented with ischemic cerebral stroke, diabetes, and obesity; 22.90% had a GLR value higher than 2, with a mean of 2.57± 2.59. No significant differences emerged for none of the comorbidities and laboratory markers presented in Table 3 and Table 4. Moreover, there were no differences in the mean value of blood sugar. The one exception was GLR, which was significantly higher when comparing the chronic patients with and without corticotherapy. However, the statistical significance was lost when comparing all the 137 non-treated patients.

## 4. Discussion

In the current study, we have compared clinical and paraclinical characteristics of 185 myasthenic patients with 895 sex- and age-matched controls, which were analyzed in the nine-year time frame considered, and included the following specific inclusion criteria. Our group of patients was characterized by a clear overall female predominance, which should be considered in the wider context of the well-established fact that autoimmune diseases are more frequent in women, with a total prevalence rate of 2 to 1 [19].

In terms of the main demographical, clinical, and paraclinical characteristics, the studied group of patients partially overlapped with the already consecrated data from the medical literature. Similar to the results of large-sample epidemiological studies performed in adult Caucasians of mainly European descent, in the present study, LOMG and GMG predominated, the latter outnumbering almost three times OMG, both in EOMG and LOMG patients. Moreover, women were the majority of the EOMG cases [5,16]. The lack of a statistically significant correlation between LOMG and men could be explained by the overall female predominance of the studied group and also by its relatively small sample size. Despite this discrepancy, the fact that men with LOMG outnumbered men with EOMG suggests that, amongst our patients, men tended to have a later MG onset than women. An indirect argument in favor of this tendency resides in the fact that the average age at admission of men with EOMG was higher by five years than that of women. While the thymic morphology related to patients’ age (including thymic atrophy) was predominant in the LOMG subgroup, in accordance with the results of other studies, several discrepancies with the literature data emerged for our group of patients: the predominance of thymomas in EOMG, thymic hyperplasia encountered almost equally in both men and women, and thymomas occurring in almost one-third of cases [5,6,16]. Possible explanations of these discrepancies with the current medical data reside in the demographical particularities of the studied patients, in which there is a clear predominance of women and of LOMG cases. In addition, we must emphasize that thymus morphology was assessed by a CT chest scan and not by an actual histological analysis.

Unlike similar studies in which the percentage of myasthenic patients with comorbidities is high but never 100%, in the current study, all of our myasthenic patients presented a minimum of one comorbid disorder [13,20,21]. This result could be explained by several particular features of the studied group: the predominance of LOMG, as it is a known fact that elderly patients accumulate comorbidities, but also the relatively prolonged disease duration in the EOMG subgroup compared to the LOMG subgroup, that could have provided the time necessary to develop additional disorders. Furthermore, we would also like to emphasize the emergency-focused profile of our clinic that might have also contributed to the 100% rate of comorbidities within our batch of patients.

Over the years, various groups reported different prevalences of autoimmune comorbidities for the myasthenic patients under study: 22.9% in the Norwegian population, but only 9.4% in the Danish population, 11.6% in the Chinese population, and 19.7% in the Japanese population [22,23,24,25]. Such differences might be explained not only by the different genetic background and particularities of each of the populations studied, but perhaps by differences in the methodology used to select the group of patients. The percentage of myasthenic patients with autoimmune comorbidities in our study group was 10.8%, and, similar to other myasthenic populations, they tended to be more frequently encountered in women and in EOMG [13,24]. In fact, all autoimmune comorbidities within the EOMG subgroup were registered only in women. The fact that the predominance of women with autoimmunities over men did not reach statistical significance should be interpreted in the key of the overall structure of our group that includes 67% women. Contrary to the previous studies according to which thymomatous MG is seldomly associated with secondary autoimmune diseases reviewed in [24], in the current study, the LOMG patients with thymoma had a slightly higher percentage of autoimmune comorbidities than the LOMG patients without thymoma. On the other hand, similar to the other studies reviewed in [24], the patients with thymic hyperplasia tended to have a higher percentage of autoimmune comorbidities. The most frequently associated autoimmune comorbidities with MG were the autoimmune thyroid diseases (15 out of 20 patients), with the highest prevalence in women with EOMG, consistent with the results published by others that reported association rates as high as almost 27% [13,24,26]. Contrary to the generally acknowledged fact that, out of the autoimmune thyroid diseases, MG tends to associate more frequently with Grave’s disease and hyperthyroidism [24,27], in our study group, chronic autoimmune thyroiditis was predominant. This unexpected high prevalence of chronic autoimmune thyroiditis might be explained, at least partially, by the retrospective cross-sectional nature of the study and also by demographic variations. Similar to other studies reviewed in [24], the vast majority of the MG patients associated with autoimmune comorbidities in our study group (95%) fell in the mild MGFA I, IIA, and IIB classes.

The association between MG and other autoimmune disorders is not surprising and it might even be considered as an additional argument in favor of MG’s autoimmune pathophysiology. Furthermore, it might also point towards a common genetic background and pathogenesis. However, the implications of the particular association between MG and autoimmune thyroid diseases go beyond the pathological mechanism, reaching the clinical manifestations, management, and outcome, as it is a known fact that hypothyroidism can aggravate myasthenic symptoms, whereas hyperthyroidism can attenuate them [28].

Despite the fact that mood disorders are amongst the most common comorbidities of neurological diseases, encountered in up to 41% of patients with MG [29], less than 25% of our myasthenic patients were registered with anxiety and depression. Similar to previous studies, anxiety was encountered more frequently than depression, both at a lower rate than the ones reported in the medical literature [29], perhaps due to the predominance of mild forms of MG (MGFA classes I and II). This low percentage was rather unexpected as the LOMG patients predominated and the higher age of MG onset is acknowledged as a risk factor for mood disorders [29]. According to a review of 32 studies dedicated to mood disorders in myasthenic patients, depression is more frequent in the more severe forms of MG, and anxiety is more frequent in MG with bulbar dysfunction, while both mood disorders are more frequent in MG with respiratory distress [29]. Nonetheless, the low percentage of recorded depression and anxiety could be due to the underdiagnosis of these mood disorders among our myasthenic patients. Data should be, thus, interpreted cautiously, as the positive early diagnosis of mood disorders in patients with MG is suboptimal worldwide, mostly due to the fact that anxiety and depression tend to be masked by the myasthenic symptoms [29]. However, an early and correct diagnosis of mood disorders is imperious in myasthenic patients as they can significantly influence mortality, morbidity, and overall quality of life, anxiety being acknowledged as a factor that precipitates the aggravation of myasthenic symptoms [29]. A patient with MG presents a high risk of entering a vicious circle in which myasthenic somatic decompensations increase the level of anxiety which, in turn, can further contribute to the aggravation of myasthenic symptoms. Furthermore, corticosteroids, used as a first-line treatment in the majority of myasthenic cases in general, can not only aggravate but also induce depression, anxiety, and psychosis, depending on duration and dose [30,31].

Both metabolic and cardiovascular diseases predominated in the elderly patients of both cases and controls, as is well-established in the medical literature. In line with this pattern, type 2 diabetes and cardiovascular diseases were higher among the LOMG subgroup, as other studies have reported as well [13,32]. However, the overall predominance of type 2 diabetes, obesity, and cardiovascular diseases in myasthenic patients, regardless of the onset age, when compared to controls was interestingly consistent with other studies and, therefore, deserves higher attention [20,21].

Regarding cardiac involvement, according to a systematic review including 41 articles focusing on cardiac involvement in MG [33], older age, and the presence of thymoma and of antibodies against muscular voltage-gated potassium channel Kv 1.4 (Kv 1.4) are risk factors for cardiomyopathy in MG patients. More specifically, 37.5% of the myasthenic patients with anti-striational antibodies (anti-titin, anti-ryanodine, and anti-Kv 1.4) develop myocarditis, which manifests as cardiac failure and/or arrythmias at 13 to 211 months from MG onset [33]. In our myasthenic patients, not only essential hypertension but also cardiac arrhythmias and heart failures were significantly more frequently encountered than in the control group. However, it is important to emphasize that, in the current descriptive retrospective study, as all comorbidities resulted from ICD codes, these rates were based on the pure co-occurrence of MG and cardiac diseases, with no details regarding the temporal association between them.

All these data suggest that the co-occurrence of MG and cardiac disease is not merely coincidental. Possible explanations might as well reside in the complex autoimmune dysregulations that characterize MG. For instance, some cardiac arrhythmias may be due to the autoimmune autonomic ganglionopathy in which autoantibodies against ganglionic AchR are present in approximately 50% of cases [33]. In MG, ganglionic AchR antibodies can appear due to the overlap between the alpha 1 subunit of muscular AchR and the alpha 3 subunit of the ganglionic AchR [33]. There is growing evidence regarding the immunopathogenic role of anti-striational antibodies, especially anti-Kv 1.4, in developing both autoimmune-mediated myocarditis and myositis in myasthenic patients. This potential cause–effect relationship is based on the grounds that some myasthenic patients have non-specific infraclinical lymphorrhages in skeletal muscles and some myasthenic patients with anti-striational antibodies and clinically manifest inflammatory myopathies have even wider-spread heart and striated muscle giant cell infiltrates with muscle fibre degeneration, which are histological findings similar to the ones in polymyositis [34]. Moreover, previous studies have shown that, out of the three anti-striational antibodies, anti-titin and anti-Kv 1.4 can be used as biomarkers for myocarditis and/or myositis in patients with MG, especially for late-onset and thymoma-associated cases [33,35].

Furthermore, possible MG-related explanations for the cardiovascular affliction reside in cytokine-mediated vasoconstriction or particular side effects of anti-myasthenic treatments [33]. Anticholinesterases exert muscarinic effects that can cause not only bradycardia or atrio-ventricular blocks, but also vasoconstriction in vessels with abnormal endothelium [33]. Moreover, high doses and long-term regimens of corticosteroids can predispose one to coronary artery diseases by inducing or aggravating hypertension and dyslipidaemia [36]. On the other hand, one can speculate that immunosuppressants may indirectly exercise a protective role against endothelial dysfunction by inhibiting tumor necrosis factor-alpha (TNF-α). This theory rests on the known fact that TNF-α secreted by the endothelial cells induces their injury by stimulating the intracellular formation of reactive oxygen species [37].

Even though cardiac diseases may sometimes be difficult to diagnose in a patient with MG as myasthenic symptoms such as fatigability and dyspnea can mask a cardiac cause, the early diagnosis of cardiac comorbidities is important because they influence not only the anti-myasthenic treatment management but also morbidity and mortality.

On the grounds that recent studies point towards a low-grade chronic systemic inflammation as a pathological background for type 2 diabetes, obesity, and cardiovascular diseases, the fact that, in the present study, both categories of diseases were encountered significantly more frequently in myasthenic patients raises the hypothesis that chronic inflammation might be the missing link [38,39,40,41]. On the other side, one must not omit the fact that some of our chronic patients’ comorbidities and elevated GLR may be involved in a cause–effect relationship with the specific anti-myasthenic treatments. Specifically, mainly the chronic administration of corticosteroids is a probable factor as it is a known fact that doses higher than 7.5 mg/day of prednisone or equivalent doses of prednisone are associated with cardiovascular risks and, to a lesser extent, mood disorders [31,42]. Moreover, corticosteroids have been acknowledged to induce metabolic and hematological side effects such as an increase in plasma glucose and circulating polymorphonuclear levels, while the levels of the other granulocytes, lymphocytes and monocytes, decrease mainly by the redistribution of these cells [43]. However, we found no significant differences in the frequency of these comorbidities and laboratory markers between patients who received chronic oral corticotherapy and patients who did not. Our findings are similar with a retrospective study that assessed the side effects of chronic oral prednsion treatment at a mean daily dose higher than in our study (36.0 mg, minimum 10 mg—maximum 50 mg) in 39 myasthenic patients and in which the most frequent side effects were increased plasma glucose level and any weight gain, while diabetes and hypertension were in a much less extent [44]. The single exception was a higher GLR mean value in our patients with corticotherapy versus chronic patients without this treatment. This finding might suggest that chronic oral corticotherapy may have influenced, to some extent, this particular parameter. However, this association did not maintain its significance within the total group without this line of treatment. Overall, these results indicate that, at least statistically, in our study group, chronic corticotherapy did not influence the number of comorbidities. We would like to stress that this study was designed as a retrospective one, the first conducted in our country, and its aim was to assess comorbidities of myasthenic patients, in order to raise awareness on the complexity of the management of this neurological autoimmune disease. The chronic inflammation theory becomes even more plausible in the context that inflammation plays a significant role in the pathogenesis of MG, as it is supported by the finding that several proteins known to be associated with inflammation (such as matrix metalloproteinase 10, transforming growth factor alpha, and extracellular newly identified receptor for advanced glycation end-products binding protein) are found to be elevated in the sera of myasthenic patients [45]. In our study, in favor of this hypothesis stands the result that the average value of GLR was higher among myasthenic patients as compared to controls, even though only the LOMG subgroup reached statistical significance in this regard. In addition, the fact that, in the LOMG subgroup, we found a significant positive correlation between GLR and TG levels and a significant similar correlation between the two age subgroups of the controls was found are highly suggestive of the presence of a low-grade inflammation correlated with a dysregulated lipid/glucose metabolism in patients with an MG onset higher than 50 years. This is consistent with other studies performed on oncological cases, which suggest that there are possible immune mechanisms linking obesity and chronic inflammation [46].

Few studies dedicated to exploring the possible relationship between low-grade inflammation and MG and to stratifying MG severity using specific combined inflammatory cells’ parameters revealed that NLR is higher in patients with MG. Hence, NLR could represent a potential prognostic marker that could be used to stratify severity and possibly predict disease activity and respiratory failure in myasthenic patients of all ages [10,47,48,49]. However, in the current study, we chose to evaluate the GLR and not the NLR in myasthenic patients versus controls for several reasons. Firstly, as neutrophils, basophiles, and eosinophils are also involved in the inflammatory process, we considered that GLR would provide a wider and a more accurate picture of a disease characterized by a chronic low-grade inflammation [50]. Secondly, given the normal independent circadian fluctuations of immune cells, with a daytime increase in granulocytes and a night-time increase in lymphocytes, the parameter that combines both main types of leucocytes would offer a much better evaluation and a better mirror of the dynamic interaction between the innate and the adaptive cellular immune responses than using a single-cell type [11]. Both innate and adaptative immune responses seem to be involved in the pathogenesis of MG, a B-cell-mediated, T-cell-dependent disease, but which is also influenced by the pro-inflammatory and immunoregulatory cytokines, some of which are also released by neutrophils and other granulocytes [2,51,52].

Overall, the relationship between the immunopathogeny of MG and chronic inflammation appears to be a complex one. On one side, the consequence of the aberrant autoimmune response in MG is chronic inflammation at the NMJ, during which the ultrastructure of its postsynaptic component is destroyed [53]. On the other side, several studies suggest that chronic inflammation in the thymus can promote MG [7]. Regardless of its histology, the thymus in MG becomes an abnormal microenvironment, with high inflammatory and autoimmune potentials that can initiate local abnormal immune responses and eventually sustain chronic inflammatory lesions at the NMJ. Thus, when referring to the immunopathogenesis of MG in relationship with thymoma, thymic atrophy, or thymic follicular hyperplasia, the concept of a “sick thymus” gains ground in the current literature [54]. From a cellular point of view, in thymic follicular hyperplasia, there is an imbalance between T-helper 17 cells and regulatory T cells, which will promote the increased production of pro-inflammatory cytokines and, therefore, chronic inflammation. In the case of thymomas, the thymic regulatory T cells are not only dysfunctional but also reduced in numbers [52]. Regarding cellularity, in thymic atrophy, similar to thymoma, the number of B cells increases. While the number of myoid and epithelial thymic cells decreases, the number of dendritic cells remains constant, however, with an increase in the expressed pro-inflammatory genes [55]. From a molecular perspective, the detection of high levels of IFN types I and III in the thymus of myasthenic patients with thymoma or thymic follicular hyperplasia suggests that a response to chronic inflammation, possibly initiated by an infection, could trigger or at least sustain autoimmunity in MG [56]. Moreover, several other pro-inflammatory cytokines such as IL-21, IFN-γ, and TNF-α are found to be increased in the thymus of myasthenic patients, which point, once again, towards chronic inflammation as an important factor in the dysfunctionality of T and generating an autoimmune response in MG [57]. Taking into consideration the fact that high concentrations of specific Toll-like receptors (TLR) such as TLR-3, TLR-4, TLR-7, and TLR-9 have been discovered in the thymus of myasthenic patients and that, through these receptors of the innate immune system, the dendritic cells mature and are capable of initiating adaptative immune responses, one can conclude that the key between chronic inflammation/infection and autoimmunity resides in the intricate interactions between the adaptive and the innate immune systems [58,59,60].

The current study encompasses a number of limitations. In the first place, we emphasize the retrospective nature of a single-center research, which included a relatively low number of subjects—however, comparable with other case–control studies.

We did not focus on the temporal association between MG and the comorbidities described. However, the aim of the study was to provide a comprehensive picture of both autoimmune and non-autoimmune comorbidities of MG in order to stress the importance of a complete pathological record in providing an adequate multi-disciplinary management of the myasthenic patient.

The thymus was assessed only by radiological methods and not by an actual histological analysis. This could represent a major drawback since the overall disparity rate between thymus imaging and histopathological analysis varies from 22% to 68% [61,62]. However, several studies state the utility of contrast CT in exploring the morphology of the anterior mediastinum [63].

Although GLR values and the inclusion of all myasthenic patients, regardless of their autoimmune and non-autoimmune comorbidities and their immunosuppressive anti-myasthenic treatment, offer a wider perspective than previous studies with restrictive exclusion criteria, they also open a path to debate. Hence, multicenter larger-size prospective studies which follow the dynamics of GLR in MG patients with and without comorbidities and immunosuppressive treatment would offer a more accurate perspective on these matters.

## 5. Conclusions

In the present case–control study, we obtained a thorough clinical and paraclinical characterization of the myasthenic population included, comprising mainly women, cases with a late onset, and generalized MG that is associated with thymoma in a higher percent than expected. Even though the encountered percent of autoimmunities was similar to other papers, the predominance of chronic autoimmune thyroiditis over Grave’s disease and hyperthyroidism was another unexpected finding, as well as the fact that all patients were associated with at least one comorbidity, autoimmune or non-autoimmune. From all comorbidities, obesity, type 2 diabetes, cerebrovascular diseases, essential hypertension, and cardiac arrythmias were significantly more frequent in myasthenic patients than in the controls. When comparing basic laboratory biomarkers between the controls and the patients with MG, the latter had higher average values of GLR, total cholesterol, and GPT. Not only were these differences more significant in the LOMG patients, but also in this particular subgroup; glucose metabolism, lipid metabolism, and liver and renal function markers were also elevated when compared to their control counterparts.

By performing not only a complete analysis of all comorbidities, both autoimmune and non-autoimmune, but also by attempting to provide potential explanations of the associations between these apparently unrelated diseases and MG, our study was a complex one dedicated to increasing awareness of the necessity of a multidisciplinary approach towards myasthenic patients. The East-European origin of the patients included in both the cases and the control groups offered a different social and cultural angle of a disease studied mainly on populations of West-European and Asian descent. This opens the path for future studies, including genetic ones which may facilitate decoding the etiology of MG.

Even though one can argue that there is no obvious connection between MG and non-autoimmune comorbidities such as endocrine, mood disorders, and metabolic and cardiovascular diseases, one must at least bear in mind that their presence in myasthenic patients contribute to the overall poor quality of life as they not only increase the burden of the disease but also can influence the therapeutical approach of MG. More specifically, some of the comorbidities (e.g., diabetes, severe cardiac failure, and recent cerebral stroke) may limit the usage of some of the conventional anti-myasthenic therapeutical classes of treatment (e.g., corticosteroids) as they can bring the risk of exacerbation. Therefore, future multicenter studies are necessary in order to follow the dynamics of associated diseases in MG patients.

## Figures and Tables

**Table 1:** Proportion of thymic radiological aspects in women and men with MG.

CT	All Cases		*p* Value for Chi2 Test
	M (*n* = 61)	F (*n* = 124)	
DET	5 (8.2%)	17 (13.7%)	0.277
RPA	43 (70.5%)	76 (61.3%)	0.221
TFM	13 (21.3%)	31 (25.0%)	0.581
*p* value for Chi2 test	*p* = 0.390		

**Table 2:** Proportion of MGFA classes in myasthenic patients with and without autoimmune comorbidities.

MGF Class	Autoimmune Diseases (*n* = 20)	Non-autoimmune Diseases (*n* = 165)	Chi2 Test *p*
I	2 (41.0%)	41 (24.8%)	0.139
IIA	7 (35.0%)	55 (33.3%)	0.882
IIB	10 (50.0%)	54 (32.7%)	0.126
IIIA	0 (0.0%)	3 (1.8%)	0.544
IIIB	0 (0.0%)	9 (5.5%)	0.286
IVA	1 (5.0%)	1 (0.6%)	0.074
IVB	0 (0.0%)	2 (1.2%)	0.622

**Table 3:** Comparisons of comorbidities and laboratory biomarkers between myasthenic patients with and without chronic oral corticotherapy (chronic cases).

Parameter	MG Cases with Oral Corticotherapy (*n* = 48)	MG Cases (Chronic + New) Without Oral Corticotherapy (*n* = 59)	Chi-Squared Test *p*
Type 2 diabetes	7 (14.60%)	10 (16.90%)	0.739
Obesity	8 (16.60%)	13 (22.0%)	0.322
Dyslipidemia	22 (45.80%)	27 (45.80%)	0.994
Hypertension	21 (43.80%)	25 (42.40%)	0.886
Ischemic stroke	11 (22.90%)	17 (28.80%)	0.489
Blood glucose > 100 mg/dL	19 (39.60%)	20 (33.90%)	0.544
Blood glucose mean ± sd	100.75 ± 28.09	96.14 ± 18.07	0.579
GLR > 2	11 (22.9%)	10 (16.90%)	0.441
GLR mean ± sd	2.57 ± 2.59	2.07 ± 0.92	0.021

**Table 4:** Comparisons of comorbidities and laboratory biomarkers between myasthenic patients with and without chronic oral corticotherapy (chronic + new cases).

Parameter	MG Cases with Oral Corticotherapy (*n* = 48)	MG Cases (Chronic + New) Without Oral Corticotherapy (*n* = 137)	Chi-Squared Test *p*
Type 2 diabetes	7 (14.60%)	26 (19.0%)	0.486
Obesity	8 (16.60%)	37 (27.0%)	0.071
Dyslipidemia	22 (45.80%)	52 (38.0%)	0.340
Hypertension	21 (43.80%)	76 (55.50%)	0.926
Ischemic stroke	11 (22.90%)	45 (32.80%)	0.190
Blood glucose > 100 mg/dL	19 (39.60%)	45 (32.80%)	0.402
Blood glucose mean ± sd	100.75 ± 28.09	98.27 ± 26.21	0.412
GLR > 2	11 (22.9%)	22 (16.10%)	0.296
GLR mean ± sd	2.57 ± 2.59	2.01 ± 0.70	0.349

## Data Availability

The data presented in this study are available on request from the corresponding author. The data are not publicly available due to privacy restrictions.

## Supplementary Materials

The following supporting information can be downloaded at: https://www.mdpi.com/article/10.3390/jcm13082273/s1, Figure S1: Age of MG and control cases. Bars represent the mean ± s.e.m. for women and men for each MG group (EOMG, LOMG) and Control (Ctrl) group (<50 years, ≥50 years) (ns = nor significant; two-tailed unpaired *t*-test).; Figure S2: Proportion of MG cases associated with the indicated ICD-10 codes. (A) Proportion of MG cases associated with the indicated mental, behavioral, and neurodevelopmental disorders. (B) Proportion of MG cases associated with the indicated diseases of the respiratory system. (C) Proportion of MG cases associated with the indicated diseases of the ear and mastoid process.
