# Supplementary material for: Autoimmune and Non-Autoimmune Comorbidities in Myasthenic Patients of East-European Descent: A Case–Control Study
Source: J Clin Med. 2024 Apr 14;13(8):2273. doi: 10.3390/jcm13082273 (PMC11051044; doi:10.3390/jcm13082273)
Supplement: Supplementary file 1 [file jcm-13-02273-s001.zip › jcm-2898797-supplementary.pdf]

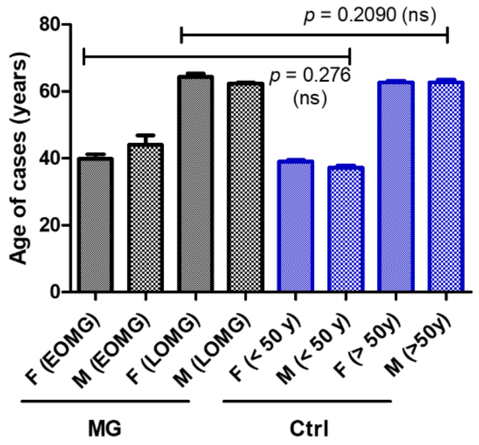

Supplementary Figure S1. Age of MG and control cases. Bars represent the mean ± s.e.m for females and males for each MG group (EOMG, LOMG) and Ctrl group (< 50 years, > 50 years) (ns = not significant; two-tailed unpaired t-test).

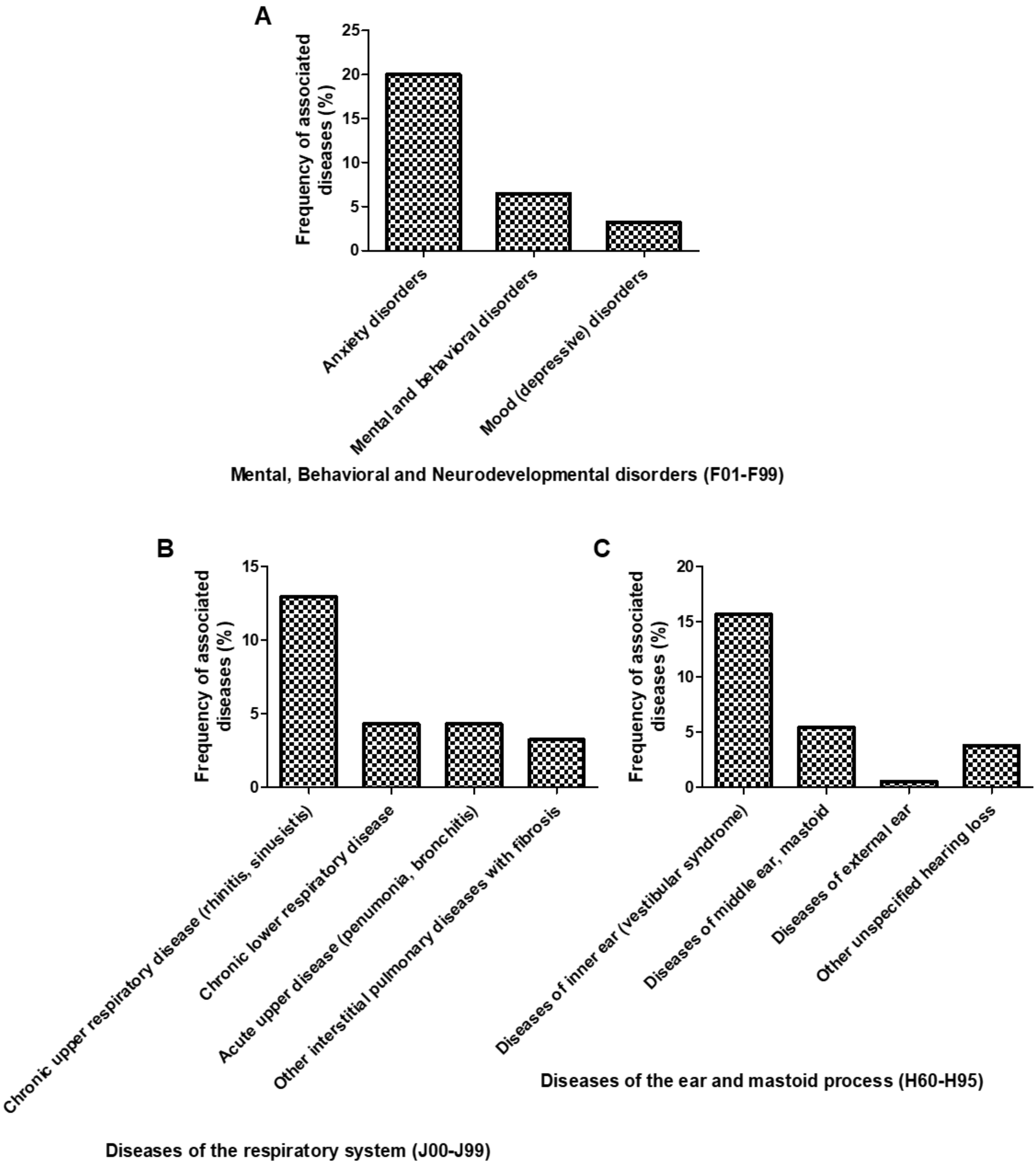

Supplementary Figure S2. Proportion of MG cases associated with the indicated ICD-10 codes.(A) Proportion of MG cases associated with the indicated mental, behavioral and neurodevelopmental disorders. **(B)** Proportion of MG cases associated with the indicated diseases of the respiratory system. **(C)** Proportion of MG cases associated with the indicated diseases of the ear and mastoid process.
